# NIAID/SMB Workshop on Multiscale Modeling of Infectious and Immune-Mediated Diseases

**DOI:** 10.1007/s11538-024-01276-2

**Published:** 2024-03-21

**Authors:** Reed S. Shabman, Morgan Craig, Reinhard Laubenbacher, Daniel Reeves, Liliana L. Brown

**Affiliations:** 1 National Institute of Allergy and Infectious Diseases, Rockville, MD 20852 USA; 2 Department of Mathematics and Statistics, Sainte-Justine University Hospital Research Centre, Université de Montréal, Montreal, Canada; 3 Department of Medicine, University of Florida, Gainesville, FL 32611 USA; 4 Department of Global Health, University of Washington, Seattle, WA 98195 USA

**Keywords:** Multiscale modeling, Collaboration, Infectious diseases

## Abstract

On July 19th, 2023, the National Institute of Allergy and Infectious Diseases co-organized a workshop with the Society of Mathematical Biology, with the authors of this paper as the organizing committee. The workshop, “Bridging multiscale modeling and practical clinical applications in infectious diseases” sought to create an environment for mathematical modelers, statisticians, and infectious disease researchers and clinicians to exchange ideas and perspectives.

## Introduction

The integration of experimental data into multiscale mathematical models can shed light on the mechanisms underlying infectious and immune-mediated diseases. The 2023 Society for Mathematical Biology (SMB) Annual Meeting provided an opportunity to convene leaders in multiscale mathematical modeling and quantitatively experimental biology, including both Ph.D. researchers and M.D. clinicians, to share their perspectives on opportunities and challenges for multiscale modeling for infectious, allergic, and immune-mediated diseases. During a five-hour event, presenters and participants sought to (1) highlight gaps, challenges, and opportunities around multiscale infectious disease (ID) models; (2) foster collaboration between mathematical modelers/statisticians and ID researchers/clinicians; (3) define what is required to incorporate multiscale models in biomedical ID applications; and (4) identify best practices and data capture conducive for multiscale modeling (booklet provided as Supplemental Information). Each of the six expert speakers were invited to share their clinical and biological outlooks on the most pressing challenges and opportunities for models to inform underlying mechanisms of and/or treatments for infectious diseases. Three sessions each featured two speakers focused on immunological, virological, and prokaryotic and eukaryotic data. The presenters defined ideal data types to answer key clinical questions and provided use-cases on ways to bring models from white-board to computer to biomedical application. After the presentations, a panel discussion featuring all speakers explored ways to initiate and achieve successful computational and biomedical collaborations.

## Workshop Summary

### Part 1. Grand Challenges for Advancing Multiscale Infectious Disease Models

*Session 1: Challenges and opportunities for modeling infectious diseases with immunological data*

Dr. Steven Kleinstein, Ph.D. (Yale University) presented “Immune Signatures of Vaccination & Infection Response” which highlighted computational methods to study the human immune responses across different vaccine platforms and pathogens. Dr. Kleinstein discussed three data-rich themes that lend themselves to modeling: (1) the breadth of the human response following influenza vaccination; (2) comparative analysis of antibody responses across different vaccine platforms; and (3) multi-omics signatures to define infection severity, such as COVID-19. Dr. Kleinstein presented work arising from several NIAID-supported programs, including the Human Immunology Project Consortium (HIPC) (NIAID 2023), a consortium that leverages systems profiling approaches to characterize the immune system in diverse populations under normal conditions and in response to vaccination and infection. HIPC has made great progress in identifying common signatures associated with successful vaccination responses (Fourati et al. 2022; Hagan et al. 2022) and has made available a compendium of vaccinology data to enable future modeling activities (Diray-Arce et al. 2022). Despite the successes he described, there remain outstanding questions that require innovative modeling methods from the molecular/cellular to single host to the population level. For example, while different vaccines can activate similar cell types and pathways, the timing of those responses can differ significantly. Further research is needed to determine the mechanisms underlying these differential kinetics. In addition, many human studies are done using blood samples, and moving from these correlative signatures to causal hypotheses remains challenging.

Dr. Kleinstein also described challenges in the field to develop standards that support data sharing and accessibility, fair and ethical secondary analysis of published data, and data integration across wide ranging cohorts and studies to enhance analysis power. The goal would be to harmonize across heterogeneous observational studies with massive amounts of data to determine signatures encompassing pathogen and host differences.

Dr. Bonnie Gunn, Ph.D. (Washington State University) discussed “Challenges and opportunities for modeling infectious diseases with immunological data: deciphering humoral immune responses to build better antibodies”. The presentation highlighted her collaborative work to define the role of antibody-mediated activation of innate immunity through interaction with the antibody Fc-receptors on immune cells (Saphire et al. 2018; Murin et al. 2022; Meyer et al. 2021; Gunn et al. 2020, 2023). She began by detailing the important secondary functions of antibodies, beyond their direct neutralization. For instance, Fc effector functions, which include antibody-dependent cellular cytotoxicity (ADCC), antibody-dependent cellular phagocytosis (ADCP), and complement-dependent cytotoxicity (CDC), may all contribute to the infection blocking or reducing power of antibodies. Through multivariate analyses that incorporate each of these different antibody functions, she demonstrated how large-scale statistical models can associate specific antibody attributes that determine the degree of protective responses. Dr. Gunn gave a striking example from her previous work characterizing antibodies from individuals who survived Ebola infection, emphasizing how her research can inform antibody engineering to enhance their efficacy against various pathogens. Dr. Gunn then discussed the potential of recombinant monoclonal antibody engineering approaches to dissect mechanisms of antibody-mediated activation of innate immune cells through the generation of an ‘Fc-effector Atlas’ (Grace et al. 2022; Gunn and Bai 2021). Through the development of an FcR atlas and systematic antibody characterization, it may be possible to accurately predict antibody efficacy across infectious diseases and engineer more effective therapeutics.

However, her work also reminds us that the precise requirements for a protective antibody response following vaccination are not yet clearly defined for Ebola, SARS-CoV-2, and other pathogens. Dr. Gunn emphasized that modeling of the immune response with defined outcomes provides an opportunity to guide ideal antigen selection and/or antigen engineering for a vaccine. Moreover, immune cell functions are dynamic and can result in either protection from infection or immune-mediated pathology. Therefore, models that incorporate these delicate feedbacks or bifurcations in dynamics could be ideal tools beyond typical statistical analysis. Indeed, models might provide pathways for optimal engineering of therapeutic antibodies with ideal neutralizing versus effector function activities. Such modeling would provide excellent opportunities for collaboration.

One specific framework she and others rely on for Ebola and SARS-CoV-2 research is “systems serology”, a suite of experimental and computational methods that can provide comprehensive (and hopefully unbiased) assessments of the humoral immune response. Yet she noted that there the borderline between fishing for associations and determining mechanistic biology depends crucially on excellent mathematical and statistical tools, as well as careful expert eyes to properly interpret observed associations. Research at this boundary is an extremely fertile ground for collaborations between clinicians, experimentalists, and modelers.

*Session 2: Challenges & opportunities modeling infectious disease data-viruses*

Dr. Catherine Gao, M.D. (Northwestern University Feinberg School of Medicine) presented, “CarpeDiem: Modeling intensive care unit electronic health record (EHR) data, focusing on patients with SARS-CoV-2 pneumonia”. She discussed how newly admitted intensive care unit (ICU) patients with similar characteristics often have different outcomes ranging from disease resolution to death. The goal of this project was to develop a method that unfolds the clinical course of an ICU patient to understand intercurrent events, with the goal of increasing the frequency of positive outcomes and reducing mortality. For this, the entirety of a patient’s medical record, and additional molecular data derived from multi-omics analyses, was used to build models of each individual patient day, amounting to over 15,000 patient ICU days with hundreds of data points for each patient. The resulting models were used to generate predictors of transitions between clinical states, which could then be used to identify potential therapeutic targets that promote transitions to more beneficial states (Gao et al. 2023).

Dr. Gao discussed how obtaining such a rich and integrated dataset was extremely challenging, requiring, in addition to significant funding, a large time commitment from her and many clinical researchers. For example, the different data types included in the models each had widely distinctive data structures. Thus, making them compatible required deep expertise and much attention to detail. Further, not all clinical courses have the same data, and a lot of critical data (and metadata) are often missing or incomplete. Missing data are frequently informative (e.g., a specific test was not ordered because the clinical progression did not necessitate it). However, this can be challenging as many methods rely on complete datasets or imputation strategies. Dr. Gao described how these challenges are compounded when clinical research is conducted at multiple clinical sites, since each can use different data formats that are not necessarily interoperable. While there has been much interest in developing EHR based models, few studies prospectively validate models (estimated at < 2% of papers) or try to implement their work, a significant limitation in this nascent field. A last complication of EHR-based modeling is that incremental modeling efforts are hindered because there are very significant barriers to approving modifications to the EHR themselves, creating a challenge to iterate and validate results. This talk illuminated a serious opportunity for mathematical and computational researchers willing to work closely with clinical researchers to bridge a crucial divide in automated data parsing and cleaning, as well as focusing on designing future studies with an eye towards eventual deployment and implementation.

Dr. Amber Smith, Ph.D. (University of Tennessee Health Science Center) presented “Modeling Viral Dynamics,” and focused on viral pneumonia caused by influenza and pneumococcal co-infection (Smith and Ribeiro 2010; Smith and Perelson 2011; Smith and McCullers 2014; Smith 2018a, 2018b; Jenner et al. 2020; Myers et al. 2021). Dr. Smith's presentation highlighted the power of mathematical modeling to integrate experimental virology and biology and to gain insights into the dynamics of viral pneumonia and other complex diseases. Several applications of modeling to biology were presented, including modeling immunopathology and tracking the heterogeneous dynamics of the immune response. Dr. Smith presented results from a mechanistic ordinary differential equations model that described links between CD8 + T cell responses, lung pathology, and disease severity in mice. Models built with data from CD8 + T cells and lung pathology were shown to be predictive of disease severity, yet there were several open questions about how to define severity (weight loss) and the best way to properly automatically estimate lung pathology.

Next, Dr. Smith presented a model to study heterogeneity of viral disease by using a virtual cohort, a recently coined modeling concept that aims to effectively perform sensitivity analyses in optimally realistic scenarios through defining “cohorts” derived from distributions observed in existing patient data. Despite all the heterogeneity considered, her results showed only two parameters in the underlying mechanistic model were required to separate severe and mild pneumonia. Another model examined bacterial pneumonia, and incorporated several variables from experimental data which were parameterized in a computational model. Dr. Smith also emphasized the importance of iterative cycling between experimentation and mathematical modeling, underscoring the importance of validation (i.e., biological confirmation) as well as parameterization of the mathematical models. She described several challenges, including secondary analyses across research groups to develop models since experimental methods are often diverse. This provides a call to action for groups to work together, especially when connecting models across scales. Further, incorporating new data into a pre-existing model is challenging because of the heterogeneity in the experimental methods, advances in the science, etc. Last, Dr. Smith referred to structural and workforce barriers to wider adoption of mathematical models in biomedical research. Specifically, model-driven experimental design requires a high level of competency in both mathematics and biology. Dr. Smith’s own career is an inspiring example of moving from a mathematics background into performing experiments in her lab, but is, for now, not a typical career path in either field.

*Challenges & opportunities modeling infectious disease data-bacteria and fungi*

Dr. Borna Mehrad, M.D. (University of Florida) presented “Modeling in invasive aspergillosis” where he highlighted a long-standing successful collaboration with his colleagues in developing a multi-scale mechanistic model of iron sequestration as part of the innate immune response to a respiratory infection with the fungus *Aspergillus fumigatus* to demonstrate that iron acquisition is critical for fungal pathogenesis, pointing the way to possible therapeutic interventions that can be studied using the model. The study was done using data from a neutropenic mouse model. The multi-scale model includes mechanistic intracellular models for macrophages, epithelial cells, as well as fungal cells, helping to clarify the role of macrophages to control and resolve fungal filaments and spores from the lung (Ribeiro et al. 2022; Oremland et al. 2016; Michels et al. 2022; Adhikari et al. 2022).

In his presentation, Dr. Mehrad argued that computational and mathematical modeling can enable scientists to develop innovative hypotheses that do not follow the more traditional approaches. He envisions their collective approach growing from the current state of the art: careful literature review and expert knowledge to design mechanistic models that then are explored to assess unobservable mechanistic consequences. While valuable, he said this approach can ultimately provide relatively ‘derivative’ and ‘safe’ hypotheses because models are often designed with hypotheses in mind. In the future, he expects less biased approaches based on large data sets like those obtained from “omics” experiments could be immediately used to expand models and lead to novel hypotheses. Yet, Dr. Mehrad posed a challenge in this case to model validation: the data requirements for iterative validation and refinement are high and need proper statistical approaches to ensure solid inferences. Taken together, there is promise that this approach to modeling allows the possibility to formulate unbiased hypotheses.

Dr. Robin Patel, M.D. asserted that, “Biologists need help.” Advances in technology are generating unprecedented amounts of data and scientists require better tools for analysis, integration and interpretation of these data. For example, advances in next generation sequencing technologies and in proteomics (e.g., MALDI-TOF MS) and their application in clinical settings are growing (Whittle et al. xxxx).

From the data generated by these technologies, processing and analytical techniques (mathematics and informatics) would benefit from improvements so that maximum clinical value and operational efficiency can be realized in medical practice and for public health.

Dr. Patel presented examples from her work, specifically highlighting the global pandemic of bacterial antimicrobial resistance (AMR) and its impact on patients in clinical settings (Boutzoukas et al. 2023). A second example focused on periprosthetic joint infection where her lab has executed multi-omics analyses of these infections. Important clinical decisions must be made in instances where patients are suffering, yet the volume and nuance of current diagnostic data are sometimes limiting.

With regards to AMR, she asked whether appropriate treatment can be mathematically predicted? A more robust genotype to phenotype pipeline would be revolutionary to help predict AMR, as well as to understand disease severity and within-host outcomes. AMR challenges and opportunities mentioned might require a hybrid of bioinformatics meets modeling and machine learning. Dr. Patel also described a gap in microbial gene annotation which is hindering analysis and presents a huge opportunity for data science approaches.

With regards to her work with periprosthetic joint infection, she commented on the need to define whether a result from data generated is actionable in a clinical setting. For example, is an identified organism a pathogen? There is a need to improve integrated mathematical approaches to properly estimate confidence in infection diagnosis and interpretation of results of predictions for clinical decisions immediately at hand. The development of analytic pipelines is needed to determine pathogen identity and clonality; phylogenomic or mathematical epidemiology is already being used to track outbreaks within healthcare settings but improvements are needed.

Finally, Dr. Patel explained that digital imaging in pathology is a data rich environment and presents an opportunity to develop better analytics and models. Digital pathology is a clinical reality, but it is in its infancy. Summing up a truly inspiring talk and session, Dr. Patel concluded by encouraging more collaborations between mathematics, computational modeling, and clinicians to both better understand disease but also to alleviate human suffering in clinical practice today.

### Part 2. Panel Discussion, Featuring the Speakers, on Future Applications of Multiscale Models (Moderated by Morgan Craig, PhD and Daniel Reeves, PhD)

A Panel Discussion with all speakers concluded the workshop with questions to all presenters.

*Question: Can you comment on common barriers and solutions for collaborations and data sharing between experimental and mathematical groups*?

*Discussion* Solutions to address barriers around data sharing often depend on effective communication and engagement across groups before initiating experiments. The community should encourage more engagement between groups to cross-fertilize across teams. Questions at the beginning of a collaborative effort can include, “what types of data do you need, and in what format?” and “what kind of data do you already have?” Moreover, identifying the right collaborator and learning terminologies across disciplines is critical. For example, “model” has a different meaning to laboratory scientists, clinicians, and mathematical modelers. One must also be willing to venture outside of one’s field and find a collaborator, and the collaborator should also be open to expand their network. It is also important to identify a common goal early on that clearly manifests the value of the collaborative efforts to all partners.

*Question: Since data sharing is potentially complex, what is the proper balance for open data sharing to protect your research project, and do you have any recommendations to achieve this balance*?

*Discussion* Data sharing takes work! But thanks to the 2023 NIH Data Management and Sharing Policy (NIH 2023), more investigators are sharing data in generalist and domain-specific repositories. It’s also important that investigators adequately resource data sharing activities since these efforts can be time consuming. Moreover, data sharing timelines need to be planned such that original investigators can be the first to describe their hard-won data while allowing that the wider community will eventually be able to use data, ideally in collaboration, to address their own scientific questions. There are additional considerations with regards to data sharing, including journal expectations, data which may result in intellectual property, and developing data and corresponding metadata so that it is FAIR (Findable, Accessible, Interoperable, and Reproducible) (Wilkinson et al. 2016).

*Question: What are the types of tools (mathematical and experimental) that you would like to see more training and development*?

*Discussion* One that rises to the top is ‘parameter estimation’, the process of computing a model's parameter values from measured data. This provides a great case study to remind modelers of the crux of reproducibility: we all must ensure that code and metadata which describes experimental and mathematical tools is shared, comprehensive, and at least somewhat readable (if not a documented package). The more work spent up front on this ultimately supports computational tool adoption and reuse.

*Question: To enable the bridging multiscale modeling to practical clinical applications, what do you recommend we teach to medical students*?

*Discussion* Specific classes in this area for medical students have the potential to accelerate progress in this area. This includes specific classes in modeling and/or multi-scale modeling, statistics, and dynamic analysis. One overarching lesson in all these classes needs to be having an open mind for testing multiple hypotheses and how to do this rigorously, or at least who to talk to when you need this kind of rigor.

*Question: How to ensure that private industry shares data when they are indirectly benefiting from public data*?

*Discussion* This is a challenging, yet important question. Funded NIH grants must comply with the NIH Data Sharing policy, which prescribes that data reside in NIH approved repositories. For contracts or Small Business Innovation Research (SBIR), there are protections around data sharing to protect intellectual property. When possible, it’s important to continue to push for open data, which is critical for model development to combat infectious diseases, allergy, and immunological diseases.

## Concluding Remarks

Our session aimed to highlight real challenges and opportunities towards using modeling on diverse biological and clinical data. Grand opportunities exist to uncover novel pathways, networks, and biomarkers, and to develop multiscale models that accurately describe and predict disease outcomes in individuals or during outbreaks, thereby informing therapeutics and vaccines. The National Institute of Allergy and Infectious Diseases (NIAID) recognizes the potential of infectious disease research that uses cutting-edge technology to generate infectious, allergic, and immunological datasets, and their application into computational multi-scale models. NIAID is committed to ensuring widespread access to all digital assets (e.g., data, analysis tools, computational models, etc.) to exploit their full value and incentivize novel discoveries. The integration of experimental data into multiscale models represents an exciting and rapidly evolving area at the forefront of biomedical research, and the 2023 SMB workshop enabled Ph.D. researchers and M.D. clinicians to share their perspectives with attendees from mathematical and computational backgrounds. By promoting collaboration across disciplines to advance multi-scale computational models, many opportunities exist to translate diverse data types into models that will assist with accurate predictions of disease severity and outcome, as well as treatment success or failure.

Challenges associated with the organization and interpretation of massive datasets and their integration into multi-scale models remain. For now, many existing models are somewhat single scale, and merging scales across collaborations is an extremely exciting yet daunting prospect for the coming years. Challenges include experimental obstacles, but also computational difficulties in effectively integrating diverse data types. Pathway/network/and biomarker discovery is immensely exciting, yet sharing raw data and insight across diverse groups can be tricky. In addition, ensuring that data is disseminated following the FAIR Data Principles represents a challenge beyond the lab, but is essential to ensure that publicly available data is reproducible and interpretable for secondary analyses by investigators worldwide. The presentations and discussions during this workshop addressed these challenges, and provided a roadmap for multidisciplinary collaborations that will generate meaningful results and informative infectious and immune-mediated disease models. Promoting rigorous interdisciplinary training, communication and collaboration across disciplines are invaluable as the field moves toward improved models to predict the impact of infectious pathogens as well as allergic and immune-mediated diseases.

Overarching takeaway lessons from the presentations and the discussions are twofold. First, while there has been some progress over the last 20 years in forming interdisciplinary collaborations between biologists and modelers, major challenges remain when trying to integrate biologists, clinicians, and mathematical modelers around projects that combine biology with clinical outcomes. Second, building and calibrating multi-scale models still presents many unsolved problems, both for mathematical modelers and for biologists and clinicians around data generation and model validation.

## Supplementary Information

Shabman, Reed (2023). Society for Mathematical Biology Annual Meeting - 2023 Workshop Booklet: Bridging multiscale modeling and practical clinical applications in infectious diseases. figshare. Conference contribution. https://doi.org/10.6084/m9.figshare.24913944.v1
